# *In Vitro* Antioxidant and Cytotoxic Activity of Some Synthetic Riparin-Derived Compounds

**DOI:** 10.3390/molecules19044595

**Published:** 2014-04-11

**Authors:** Geandra B. L. Nunes, Paola R. Policarpo, Luciana M. Costa, Teresinha G. da Silva, Gardenia Carmen G. Militão, Celso A. Câmara, José Maria Barbosa Filho, Stanley Juan C. Gutierrez, Mohammed T. Islam, Rivelilson M. de Freitas

**Affiliations:** 1 Laboratory for Research in Experimental Neurochemistry, Campus Ministro Petrônio Portella, Federal University of Piauí, Teresina, PI 64049550, Brazil; E-Mails: geandraenf@hotmail.com (G.B.L.N.); paolla_dallet@hotmail.com (P.R.P.); luciana.muratori@hotmail.com (L.M.C.); 2 Laboratory of Bioassays for Drug Research, Federal University of Pernambuco, Recife, PE 50670-901, Brazil; E-Mails: teresinha.goncalves@ufpe.br (T.G.S.); gcgadelha@yahoo.com.br (G.C.G.M.); 3 Department of Chemistry, Federal Rural University of Pernambuco, Recife, PE 52171900, Brazil; E-Mail: ccelso@dcm.ufrpe.br; 4 Laboratory of Pharmaceutical Technology Federal University of Paraíba, João Pessoa, PB 58051-900, Brazil; E-Mail: jbarbosa@ltf.ufpb.br; 5 Laboratory Chemistry of Bioactive Natural and Synthetic Products, Federal University of Piauí, Teresina, PI 64049550, Brazil; E-Mail: stanleychavez@yahoo.com.br; 6 Department of Pharmacy, Faculty of Science and Engineering, Southern University Bangladesh, Mededibag, Chittagong-4000, Bangladesh; E-Mail: mti031124@gmail.com

**Keywords:** antioxidant activity, cytotoxicity, riparins

## Abstract

This study aimed to study the *in vitro* antioxidant activity and cytotoxicity on tumor cells lines of six synthetic substances derived from riparins. All the substances showed antioxidant activity and riparins C, D, E, F presented cell growth inhibition rates greater than 70%, suggesting that these molecules have antitumor properties. These substances also caused greater than 80% releases of cytoplasmic lactate dehydrogenase enzyme (LDH). Although the antioxidant and antitumor properties presented herein require further assessment, the outcomes indicate that these novel riparins are promising biologically active compounds.

## 1. Introduction

The synthesis of new substances with pharmacological activity a great challenge frequently approached by the conversion of medicinal plant products into medicines. Historical experience with natural products as therapeutic agents has evolved into sophisticated isolation of active chemical entities from ethnopharmacological plants, and in modern medicine, natural products are still increasingly the primary sources in early drug discovery [1]. The *Lauraceae* family contains an expressive number of species with great diversity of medicinal and industrial uses and a high commercial value, which has led to its increasing exploitation, making this family vulnerable to extinction [2]. Some alkaloids of the alkamide group were isolated from the green fruit of *Aniba riparia* (Nees) Mez, a typical *Lauraceae* plant of the Amazon Region. Its alkaloids demonstrated pharmacological activity in preclinical trials and less side effects than the classical medicines with the same therapeutic use [3].

In using natural bioactive products as the basis to the development of new drugs, the industry usually faces the challenge of the low concentrations of selected substances present in natural sources, which generally makes their commercial exploitation unfeasible. However, the synthesis of these substances and their derivatives frequently allows the pharmacophore to be established and modulation of biological profiles, representing an excellent opportunity to explore the biological actions of these synthetic and semi-synthetic organic compounds [4].

The response to this increasing demand for structurally innovative substances for pharmacological evaluation has established a new paradigm in the search for prototype compounds and in the optimization/development of pre-existing ones, recognizing sustainable use of the Brazilian biodiversity and development of a national industry and ensuring access and appropriate usage of medicinal plants, phytotherapics and their analogues by the population [5].

Natural alkamides constitute a special class of alkaloids containing amide functions that is restricted in Nature to a few representatives [6]. The biological activities mentioned in the literature and attributed to extracts of fruit and the calyx of *A*. *riparia* [2], awakened interest in verifying the pharmacological potential of these amides, which were isolated and synthetized by the first time at the Laboratory of Pharmaceutical Technology of Federal University of Paraiba [6,7]. These amides were named riparins as a tribute to the species studied [8].

**Scheme 1:**
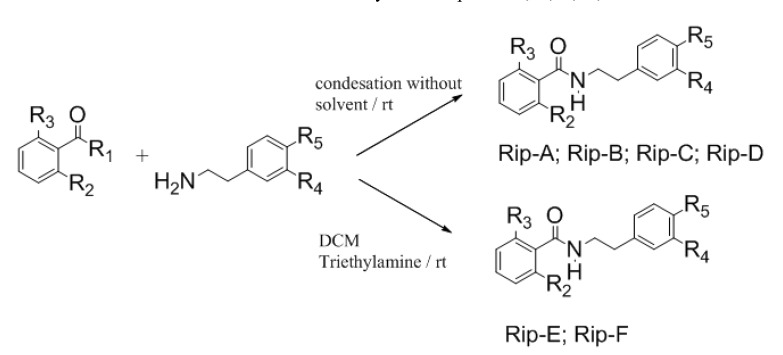
General reaction of synthetic riparins A, B, C, D, E and F.

Rip-A: R_1_ = Cl; R_2_ = R_3_ = R_4_ = R_5_= H; Rip-B: R_1_ = Cl; R_2_ = R_3_ =H; R_4_ = R_5_= OCH_3_; Rip-C: R_1_ = OCH_3_; R_2_ = OH; R_3_ = R_4_ = R_5_= H; Rip-D: R_1_ = OCH_3_; R_2_ = OH; R_3_ = H; R_4_ = R_5_= OCH_3_; Rip-E: R_1_ = OCH_3_; R_2_ = R_3_ = OH; R_4 _= R_5_= H; Rip-F: R_1_ = OCH3; R_2_ = R_3_ =OH; R_4_ = R_5_= OCH_3_

Using the Schotten Bauman reaction, some riparin derivatives were prepared and named riparins A and B, and by the condensation of the corresponding metallated esters with substituted phenethylamines, riparins C, D, E and F were obtained (Scheme 1). These analogues were obtained using a simple and reliable method that makes feasible the commercial exploitation of these molecules by the pharmaceutical industry in face of the low availability of the selected substance in natural sources [9].

Free radicals and reactive oxygen species are involved in several pathological and physiological processes, such as epileptic seizures, inflammation, and cancer [10]. Many products have antioxidant activity, which can be important in the establishing their therapeutic properties.

Oxidative stress processes occur due to the existence of an imbalance between oxidant and antioxidant compounds, favoring the excessive generation of free radicals or due to their reduced rate of removal. These processes lead to biomolecule oxidation that in humans causes a series of cell disorders such as lipid peroxidation, protein and enzymatic damage, as well as DNA alterations, which can be related to many deleterious processes such as cancer, atherosclerosis, diabetes mellitus, rheumatoid arthritis, muscular dystrophy, cataract, neurological disorders, respiratory diseases and premature aging [11,12], manifested by potential the oxidative damage of cells and tissues [13].

The balance between the production of reactive oxygen species (ROS) and antioxidant defenses determines the degree of oxidative stress [14]. There is significant evidence that generation of reactive oxygen species and the corresponding responses to oxidative stress are fundamental factors that determine, or at least influence the longevity process [15]. Antioxidants, besides their traditional use as food additives to protect from deterioration and as stabilizers in fuels and lubricants to avoid oxidation, have been proposed as therapeutic tools to combat health damages caused by oxidative stress [16].

This research evaluated the antioxidant potential of several synthetized riparins, by measuring TBARS content, hydroxyl radical levels and nitrite ion formation. In a second step, the effective inhibitory concentration (EC_50_) values of the riparins against the formation of free radicals were also calculated in order to compare them with known antioxidants. The cytotoxic effects of the riparins on the viability of three tumor cells lines were also evaluated by MTT colorimetric assays and cytoplasmic enzyme lactate dehydrogenase (LDH) level measurements in order to screening their antitumor potential.

## 2. Results and Discussion

After their synthesis, the riparins Rip A-F were identified and their structures elucidated by information acquired by ^1^H-NMR and ^13^C-NMR, respectively, (Table 1 and Table 2).

**Table 1:** ^1^H-NMR data of riparin derivatives (Rip-A, -B, -C, -D, -E and -F) in DMSO-*d_6_*.

H	Rip-A	Rip-B	Rip-C	Rip-D	Rip-E	Rip-F
1	-	-	-	-	-	-
2	7.81 (dd 2.0;8.0)	7.82 (dd 1.8;7.8)	-	-	-	-
3	7.45(m)	7.45(m)	7.28(m)	6.86(t 7.6)	7.24(m)	6.33(d 8.4)
4	7.45(m)	7.45(m)	7.28(m)	6.86(t 7.6)	7.24(m)	7.13(t 8.2)
5	7.45(m)	7.45(m)	7.28(m)	7.38(t 7.0)	7.24(m)	6.33(d 8.4)
6	7.81 (dd 2.0;8.0)	7.82 (dd 1.8;7.8)	7.66 (dd 1.4;8.1)	7.83 (d 7.6)	-	-
1'	-	-	-	-	-	-
2'	7.24(m)	6.81 (s)	7.28(m)	6.8(s)	6.35(d 8.0)	6.81 (s)
3'	7.24(m)	-	7.28(m)	-	7.24(m)	-
4'	7.24(m)	-	6.872 (t7.8)	-	7.24(m)	-
5'	7.24(m)	6.84(d8.4)	7.28(m)	6.8(d)	7.24(m)	6.75(d 8.0)
6'	7.24(m)	6.73(dd2;8.0)	7.28(m)	6.73(dd1.8;8.2)	6.35(d 8.0)	6.86(d 8.0)
7'	2.83(t 7.8)	2.77(t 7.6)	2.86(t 7.8)	2.78(t 7.40)	2.84(t7.4)	2.77(t 7.2)
8'	3.47(q 6.8)	3.46(q 7.6)	3.52(q 6.8)	3.50( q 6.8)	3.58(q 6.8)	3.56(q 7.4)
N-H	8.57(sl)	8.552(s)	8.93(t 5.4)	8.87(t 5.6)	8.99(t 4.8)	8.9(sl)
2-OH	-	-	12.628 (s)	12.59(s)	12.6(s)	12.58(s)
6-OH	-	-	-	-	12.6(s)	12.58(s)
3'-OMe	-	3.69 (s)	-	3.69(s)	-	3.7(s)
4'-OMe	-	3.69 (s)	-	3.69(s)	-	3.7(s)

**Table 2:** ^13^C-NMR data of riparin derivatives (Rip-A, -B, -C, -D, -E and -F) in DMSO-*d_6_*.

	Rip-A	Rip-B	Rip-C	Rip-D	Rip-E	Rip-F
1	134.64	134.67	115.26	115.33	102.50	102.50
2	127.11	127.06	160.03	159.99	160.23	160.23
3	128.65	128.19	117.37	117.37	107.17	107.18
4	131.04	132.02	133.60	133.60	133.32	133.34
5	128.65	128.19	118.54	118.55	107.17	107.18
6	127.11	127.06	127.67	127.71	160.23	160.23
1'	139.54	130.99	139.24	131.70	139.14	131.52
2'	128.24	112.61	128.63	112.55	128.44	112.48
3'	128.32	148.62	128.36	148.64	128.66	148.70
4'	126.07	147.25	126.17	147.31	126.28	147.34
5'	128.32	111.97	128.36	111.94	128.66	111.92
6'	128.24	120.48	128.63	120.50	128.44	120.49
7'	35.13	34.60	34.85	34.37	34.77	34.33
8'	40.90	41.01	40.54	40.69	40.19	40.29
N-H	-	-	-	-	-	-
2-OH	-	-	-	-	-	-
6-OH	-	-	-	-	-	-
3'-OMe	-	55.51	-	55.49	-	55.48
4'-OMe	-	55.33	-	55.32	-	55.33
C=O	166.18	166.15	168.87	168.79	170.00	169.95

The analogues, like the natural riparins, were subjected to pharmacological screening in order to determine their pharmacological actions and the structure-activity relationship of this class of compounds.

Initially, the tests performed were the screening of *in vitro* antioxidant activity and cytotoxicity against selected tumor cells lines. Table 3 presents the *in vitro* antioxidant potential of riparins A-F measured in TBARS production, hydroxyl radical content and nitric oxide formation assays.

The results also permitted the calculation of the inhibitory concentration (IC_50_) in μg/mL for each riparin evaluated (Rip A–F), in each of the different tests, confirming *in vitro* antioxidant potential of these substances (Table 4).

**Table 3:** Antioxidant potential *in vitro* of riparins A-F in TBARS production, hydroxyl radical content and nitric oxide formation.

Antioxidant tests				μg/mL	Rip-A	Rip-B	Rip-C	Rip-D	Rip-E	Rip-F
Nitrite Production (% induced by SNP)				1	75.1 ± 1.31 ^a^	75.8 ± 1.02 ^a^	69.3 ± 1.07 ^a^	72.6 ± 1.81 ^a^	65.9 ± 1.30 ^a^	74.9 ± 2.27 ^a^
				5	29.1 ± 0.61 ^a^	34.1 ± 1.85 ^a^	34.9 ± 0.23 ^a^	32.2 ± 1.45 ^a^	37.7 ± 0.95 ^a^	35.3 ± 0.96 ^a^
Vehicle ^1^	System	Trolox (140 μg/mL)		10	29.2 ± 2.05 ^a^	29.1 ± 2.00 ^a^	22.9 ± 1.31 ^a^	28.9 ± 1.74 ^a^	25.1 ± 1.37 ^a^	21.5 ± 0.98 ^a^
34.88 ± 0.96	100.4 ± 2.01 *	40.2 ± 2.80 ^a^		25	24.3 ± 0.62 ^a^	23.1 ± 0.97 ^a^	19.8 ± 0.83 ^a^	24.7 ± 1.77 ^a^	20.1 ± 0.71 ^a^	19.9 ± 0.71 ^a^
				100	20.4 ± 1.20 ^a^	20.6 ± 0.98 ^a^	16.5 ± 1.44 ^a^	17.3 ± 2.38 ^a^	15.4 ± 1.11 ^a^	17.3 ± 0.22 ^a^
2-Deoxyribose degradation (%)				1	97.1 ± 0.78 ^a^	98.4 ± 0.39 ^a^	95.5 ± 1.30 ^a^	93.5 ± 1.17 ^a^	79.9 ± 0.64 ^a^	83.3 ± 2.13 ^a^
				5	76.9 ± 1.10 ^a^	79.8 ± 1.34 ^a^	80.9 ± 1.29 ^a^	67.9 ± 1.74 ^a^	62.2 ± 2.14 ^a^	61.5 ± 0.98 ^a^
Vehicle^1^	System		Trolox (140 μg/mL)	10	54.5 ± 0.85 ^a^	58.5 ± 0.85 ^a^	58.7 ± 1.37 ^a^	56.9 ± 0.82 ^a^	53.4 ± 1.79 ^a^	57.6 ± 1.47 ^a^
13.96 ± 0.96	100.2 ± 1.35 *		21.4 ± 0.95 ^a^	25	49.6 ± 0.89 ^a^	47.7 ± 0.44 ^a^	52.1 ± 0.13 ^a^	51.9 ± 1.27 ^a^	51.4 ± 1.47 ^a^	55.1 ± 1.78 ^a^
				100	44.8 ± 0.30 ^a^	47.2 ± 0.43 ^a^	46.7 ± 0.35 ^a^	47.3 ± 0.88 ^a^	49.4 ± 0.85 ^a^	50.9 ± 2.33 ^a^
TBARS levels (% AAPH)				1	40.9 ± 1.77 ^a^	44.5 ± 1.31 ^a^	39.3 ± 0.60 ^a^	41.2 ± 0.96 ^a^	41.4 ± 0.69 ^a^	38.8 ± 0.99 ^a^
				5	38.1 ± 0.49 ^a^	40.4 ± 0.85 ^a^	38.8 ± 1.07 ^a^	39.4 ± 0.71 ^a^	38.6 ± 0.63 ^a^	35.9 ± 0.41 ^a^
Vehicle^1^	System		Trolox (140 μg/mL)	10	36.7 ± 0.66 ^a^	38.3 ± 1.05 ^a^	34.3 ± 0.92 ^a^	37.3 ± 0.68 ^a^	35.5 ± 0.37 ^a^	35.2 ± 0.44 ^a^
18.38 ± 1.84	99.90 ± 1.32 *		55.3 ± 6.48 ^a^	25	35.1 ± 0.88 ^a^	36.5 ± 1.21 ^a^	29.5 ± 1.91 ^a^	34.4 ± 0.76 ^a^	31.3 ± 0.76 ^a^	34.6 ± 0.49 ^a^
				100	25.8 ± 0.48 ^a^	29.7 ± 1.31 ^a^	25.7 ± 2.62 ^a^	25.6 ± 0.66 ^a^	28.2 ± 0.39 ^a^	30.3 ± 0.47 ^a^

* *p <* 0.05, compared to system (ANOVA and *t* -*S*tudent-Newman-Kewls as *post hoc* test); ^a^ *p <* 0.05, compared to vehicle (ANOVA and *t*-Student-Newman-Kewls as *post hoc* test); ^1^ Vehicle: 0.05% Tween 80 dissolved in 0.9% saline.

**Table 4:** IC_50_ of *in vitro* antioxidant potential of riparins A, B, C, D, E and F in TBARS production, (OH) hydroxyl radical content and nitrite formation.

Riparins	Riparin A			Riparin B			Riparin C		
**Parameters**	**OH radical**	**Nitrite**	**TBARS**	**OH radical**	**Nitrite**	**TBARS**	**OH radical**	**Nitrite**	**TBARS**
**IC**_50_ **(μg/mL)**	1.501	0.8404	1.147	1.639	0.7948	1.032	0.9516	0.7240	0.8971
**CI**	0.81–2.77	0.41–1.69	0.59–2.20	0.82–3.24	0.40–1.57	0.57–1.86	0.47–1.89	0.37–1.40	0.49–1.63
**r2**	0.84	0.80	0.83	0.80	0.81	0.85	0.81	0.81	0.85
**Riparins**	**Riparin D**			**Riparin E**			**Riparin F**		
**Parameters**	**OH radical**	**Nitrite**	**TBARS**	**OH radical**	**Nitrite**	**TBARS**	**OH radical**	**Nitrite**	**TBARS**
**IC**_50_ **(μg/mL)**	1.358	1.270	0.9958	1.054	0.5224	0.7821	1.016	0.5817	0.6231
**CI**	0.68–2.71	0.69–2.31	0.55–1.80	0.55–1.99	0.28–0.94	0.45–1.33	0.56–1.81	0.32–1.03	0.37–1.04
**r**^2^	0.81	0.85	0.85	0.83	0.83	0.87	0.86	0.84	0.87

IC_50_: inhibitory concentration 50% in μg/mL; CI: Confidence Interval in μg/mL; r^2^: coefficient of determination.

The TBARS level quantification showed that riparins A-F exert a significant antioxidant action against peroxyl radicals at all concentrations tested, protecting lipids from oxidation (Table 3). A similar result was obtained with Trolox (140 μg/mL), a synthetic hydrophilic analogue of α-tocopherol, which is widely used as a standard antioxidant. The substances also sequestered nitric oxide (NO), since there was a significant decrease in the production of that compound caused by the riparins at all concentrations tested (Table 3). The antioxidant activities *in vitro* demonstrated in the riparin tests can be exploited in a possible *in vivo* protection of biomolecules, against damage caused by free radicals [17].

The mechanism of action to the antioxidant activity can be theoretically suggested from the chemical structures of the substances. Riparins A and B have, structurally, a greater free radical capture potential and, consequently, a greater potential for antioxidant activity, which is related to the presence of hydrogen with little stabilization in their molecules, enabling the capture by free radicals and turning them inactive. There was no difference of antioxidant activity among the riparins tested, suggesting that the hydrogens bonded to the nitrogen of riparins C, D, E and F, despite being stabilized through hydrogen bonds with hydroxyl radicals, still retain their antioxidant capacity.

The search for antioxidant molecules reflects the interest of researchers in detecting compounds isolated from medicinal plants that present that property and it reinforces the importance of identification of natural and/or synthetic compounds with this potential. Previously, the ethanol extracts of medicinal plants from the semiarid Piauí region (*Terminalia brasiliensis* Camb., *Terminalia fagifolia* Mart. et Zucc., *Cenostigma macrophyllum* Tul. var. *acuminata* Teles Freire, *Qualea grandiflora* Mart.), did not show differences in their potential to scavenge free radicals [18], while the riparins, besides their low availability, have demonstrated promising bioactivities in *in vitro* antioxidant tests and to bioprospect pharmacological products that promote protection against cell disorders caused by oxidative stress.

Table 5 shows the cytotoxicity of riparins (IC_50_) upon three cancer cells lines: HL-60 (pro-myelocytic leukemia), NCIH-292 (lung carcinoma), and HEP-2 (laryngeal carcinoma).

**Table 5:** IC_50_ values and confidence intervals in µg/mL for riparins A, B, C, D and F on tumor cell lines.

Riparins	NCIH-292	HEP-2	HL-60
**Rip-A**	>25	>25	>25
**Rip-B**	>25	>25	>25
**Rip-C**	>25	7.3 (5.2–10.1)	3.3 (2.0–5.6)
**Rip-D**	>25	7.3 (5.4–9.9)	9.0 (6.8–11.9)
**Rip-E**	>25	nt	1.9 (1.5–2.4)
**Rip-F**	10.3 (7.6–13.8)	7.8 (4.5–13.5)	11.4 (9.1–14.3)

nt: not tested.

Riparins C, D, E and F revealed cytotoxic action on neoplastic cells (leukemia and lung and laryngeal carcinoma), with IC_50_ values ranging from 1.9 to 11.4 µg/mL. This activity was more evident on HL-60 cells. To analyze the cytotoxic potential of riparins A-F they were also tested (at 25 µg/mL) to determine the percentage of the release of LDH. This measurement is used as a parameter to evaluate cellular or tissue damage (Table 6).

**Table 6:** Percentage of cytoplasmatic enzyme LDH releasing induced by riparins at concentration of 25 µg/mL on murine peritoneal macrophages RAW 264.7.

Riparins	LDH	
	% Release	Deviation
**Negative Control**	3.13	0.68
**Rip-A**	28.13	3.02 ^a^
**Rip-B**	17.31	1.71 ^a^
**Rip-C**	87.35	2.37 ^a^
**Rip-D**	83.12	1.04 ^a^
**Rip-E**	96.91	0.15 ^a^
**Rip-F**	91.01	0.19 ^a^
**Triton X-100 1%**	99.96	1.21 ^a^

^a^ *p <* 0.05, when compared with negative control (ANOVA and Tukey as *post hoc* test).

Studies evaluating the cytotoxicity and antitumor activity of substances derived from natural products are numerous. *Tabernaemontana catharinensis* A. is a medicinal plant that, like *A*. *riparia*, produces alkaloids, among them heyneanine, coronaridine and voacangine, which have had their cytotoxic activity tested on human tumor cell lines, including the HEP-2 line. Coronaridine was the one which exhibited the greatest cytotoxic activity on the larynx carcinoma cell line HEP-2 (IC_50_ = 54.47 µg/mL) compared to the other alkaloids tested (voacangine IC_50_ = 159.33 µg/mL and heyneanine IC_50_ = 689.45 µg/mL) [19]. The study mentioned reinforces the merit and the necessity of the investigation of coronaridine as a possible antitumor agent, in this context, the riparins present more promising bioactivity, presenting IC_50_ for same strain of tumor cells (HEP-2) up to 7.3 µg/mL.

Similar to the cell viability outcomes with MTT, In LDH assays the riparins C, D, E and F presented cytotoxicity on RAW 264.7 macrophages and showed LDH release levels similar to those obtained with the positive control (Triton X-100 1%) (Table 6). Taken together, these discoveries (cytotoxicity and antioxidant activity) support the antitumor of the compounds.

## 3. Experimental

### 3.1. General Information

The reagents and solvents used in preparation of riparins and tests were obtained as follows: hexane, methanol, 2-phenylethylamine, 3,4-dimethoxyphenethylamine, methyl salicylate, 2,6-dihydroxybenzoic acid, dichloromethane, phosphoric acid 4%, trichloroacetic acid, AAPH, RPMI 1640 medium (Sigma-Aldrich, St. Louis, MO, USA.); triethylamine, benzoyl chloride, dichloromethane, thiobarbituric acid, NaOH, HCL, ethyl acetate, sodium nitroprusside, Griess reagent, glutamine (Merck, Darmstadt, Germany). Compounds were purified by column chromatography using a vertical glass column (silica gel 60 (SiO_2_) 70~230 Mesh; SysCroma, Santa Maria, RS, Brazil) and a mixture of organic solvents in increasing order of polarity (hexane-hexane/dichloromethane (1:1)-dichloromethane and dichloromethane/methanol 90:10). Melting points were determined on a MQAPF-302 digital apparatus model manufactured by Microquímica (Palhoça, SC, Brazil). Hydrogen (^1^H-NMR) and carbon (^13^C-NMR) nuclear magnetic resonance spectra were obtained on Bruker spectrometers (Avance DPX-300 and Avance DRX-500), operating at 300 or 500 MHz for ^1^H and 75 or 125 MHz for ^13^C, respectively.

### 3.2. Compound Synthesis

Rip A-F were prepared using the Schotten-Bauman reaction methodology described previously in the literature [9,20].

#### 3.2.1. *N-Phenethylbenzamide* (Rip-A)

Benzoyl chloride (3.5 mmol) and 2-phenylethylamine (7.0 mmol) were mixed. The reaction mixture without solvent was left shaking for 30 min at room temperature. After purification by column chromatography N-phenethylbenzamide (Rip-A, 0.68 g, 84% yield) was obtained. Melting point: 115 °C [21]. ^1^H and ^13^C-NMR date: see Table 1 and Table 2.

#### 3.2.2. *N-[2-(3,4-Dimethoxyphenyl)ethyl]benzamide* (Rip-B)

Benzoyl chloride (3.5 mmol) and 3,4-dimethoxyphenethylamine (7.0 mmol) were reacted for 30 min to afford N-[2-(3,4-dimethoxyphenyl)ethyl]benzamide (Rip-B, 0.82 g, 80% yield). Melting point: 90 °C [22]. ^1^H and ^13^C-NMR date: see Table 1 and Table 2.

#### 3.2.3. *2-Hydroxy-N-phenethylbenzamide* (Rip-C)

Methyl salicylate (3.3 mmol) and phenylethylamine (6.5 mmol) were shaken for 8 h at room temperature to give 2-hydroxy-N-phenethylbenzamide (Rip-C, 0.52 g, 65% yield). Melting point: 95 °C [23]. ^1^H and ^13^C-NMR date: see Table 1 and Table 2.

#### 3.2.4. *2-Hydroxy-N-[2-(3,4-methoxyphenyl)ethyl]benzamide* (Rip-D)

Methyl salicylate (3.3 mmol) and 3,4-dimethoxyphenethylamine (6.6 mmol) were mixed at room temperature under magnetic stirring for 6 hours to give 2-hydroxy-N-[2-(4-methoxyphenyl)ethyl]-benzamide (Rip-B, 34% yield). Melting point: 96 °C [23]. 1H and 13C-NMR date: see Table 1 and Table 2.

#### 3.2.5. *2,6-Dihydroxy-N-phenethylbenzamide* (Rip-E)

Phenylethylamine (5.9 mmol) was added to a solution of 2,6-dihydroxybenzoic acid ester (2.9 mmol) in dichloromethane (30 mL) containing triethylamine (7.0 mmol) and shaken for 5 h. The mixture then was neutralized with 2% HCl. Next, three portions of 20 mL each were extracted with ethyl acetate (100 mL). The organic phases were combined, dried with anhydrous sodium sulfate, filtered and concentrated under vacuum to afford 6-didyhroxy-N-phenethylbenzamide (Rip-E, 0.58 g, 75% yield). Melting point: 155 °C [24]. 1H and 13C-NMR date: see Table 1 and Table 2.

#### 3.2.6. *N-[2-(3,4-Dimethoxyphenyl)ethyl]-2,6-dihydroxybenzamide* (Rip-F)

3,4-Dimethoxyphenethylamine (5.9 mmol) was added to a solution of methyl 2,6-dihydroxy-benzoate (2.9 mmol) in dichloromethane (30 mL) containing triethylamine (7,0 mmol) and shaken for 5 h. The mixture was neutralized with 2% HCl and extracted with three portions of ethyl acetate. The organic phases were combined, dried with anhydrous sodium sulfate, filtered and concentrated on a rotary evaporator, to give *N*-[2-(3,4-dimethoxyphenyl)ethyl]-2,6-dihydroxybenzamide (Rip-F; 0.66 g; 69.0% yield). Melting point: 167 °C. ^1^H and ^13^C-NMR date: see Table 1 and Table 2.

### 3.3. In Vitro Antioxidant Potential Tests

For the *in vitro* antioxidant tests the samples were emulsified in 0.05% Tween 80 dissolved in saline 0.9% (used as the vehicle) and tested at concentrations of 1, 5, 10, 25 and 100 µg/mL. The samples for the cytotoxicity tests were diluted in pure and sterile DMSO and tested at concentrations of 25 µg/mL.

#### 3.3.1. Hydroxyl Radical Scavenging Activity

The formation of OH radicals from Fenton reagents was quantified using the condensation product of the 2-deoxyribose oxidative degradation product, malonaldehyde, with 2-thiobarbituric acid (TBA) [25]. Briefly, typical reactions were started by the addition of Fe^2+^ (FeSO_4_ 6 mM final concentration) to solutions containing 5 mM 2-deoxyribose, 100 mM H_2_O_2_ and 20 mM phosphate buffer (pH 7.2). In order to determine the antioxidant activity of the riparins against the hydroxyl radical, individual tests were performed on each one of the six substances, in which different concentrations of the riparins were added to the system before Fe^2+^ addition (Rip-A; Rip-B; Rip-C; Rip-D; Rip-E and Rip-F). Reactions were carried out for 15 min at room temperature and were quenched by the addition of phosphoric acid 4% (*v/v*), followed by TBA 1% (thiobarbituric acid, *v/v* in 50 μL NaOH). The solutions were boiled for 15 min to 95 °C and then cooled at room temperature. The absorbance was measured at 532 nm and the results were expressed as MDA equivalents formed by Fe^2+^ and H_2_O_2_ [25].

#### 3.3.2. Evaluation of the Antioxidant Potential of Riparins against the Formation of TBARS

The TBARS assay was employed to quantify lipid peroxidation and an adapted TBARS method was used to measure the antioxidant capacity of the riparins (Rip-A, -B, -C, -D, -E and -F) using egg yolk homogenate as lipid rich substrate [26]. Egg yolk was homogenized (1% *w/v*) in 20 mM phosphate buffer (pH 7.4), 1 mL of homogenate was sonicated and then homogenized with 0.1 mL of the samples at concentrations of 1, 5, 10, 25 and 100 µg/mL. Lipid peroxidation was induced by the addition of 0.1 mL of 2,2'-azobis(2-methylpropionamidine) dihydrochloride solution (AAPH; 0.12 M). Control was evaluated with only the vehicle. Reactions were carried out for 15 minutes at 37 °C. After cooling, samples (0.5 mL) were centrifuged with 0.5 mL of trichloroacetic acid (15%) at 1,200 *×g* for 10 min. An aliquot of 0.5 mL from supernatant was mixed with 0.5 mL TBA (0.67%) and heated at 95 °C for 30 min. After cooling, sample absorbance was measured using a spectrophotometer at 532 nm. The results were expressed as percentage of TBARS formed by AAPH alone (induced control).

#### 3.3.3. Scavenging Activity of Nitric Oxide (NO)

Nitric oxide was generated from spontaneous decomposition of sodium nitroprusside in 20 mM phosphate buffer (pH 7.4). Once generated, NO interacts with oxygen to produce nitrite ions, which are measured by the Griess reaction [27]. The reaction mixture (1 mL) containing 10 mM sodium nitroprusside (SNP) in phosphate buffer and the riparins at various concentrations of 1, 5, 10, 25 and 100 µg/mL were incubated at 37 °C for 1 h. A 0.5 mL aliquot was taken and homogenized with 0.5 mL Griess reagent. The absorbance of chromophore was measured at 540 nm. Percent inhibition of generated nitric oxide was measured by comparing the absorbance values of negative controls (only 10 mM sodium nitroprusside and vehicle) to assay preparations. Results were expressed as percentage of nitrite formed by SNP alone.

### 3.4. Cytotoxicity against Tumor Cell Lines

Cells were maintained at 37 °C with 5% CO_2 _in RPMI 1640 (Himedia^TM^, Mumbai, India) or DMEM medium (Gibco^TM^, Grand Island, NY, USA) supplemented with 2 mM glutamine, 100 U/mL penicillin, 100 μg/mL streptomycin. The antiproliferative activities of riparins were evaluated in the following human cancer cells lineage: HL-60 (pro-myelocytic leukemia), NCIH-292 (lung carcinoma), and HEP-2 (laryngeal carcinoma) obtained from the Rio de Janeiro Cell Bank (RJ-Brazil). For all experiments, 100 µL of tumor cells were plated in 96-well plates (6 × 10^4^ cells/cm^2^ for adherent cells or 9 × 10^4^ cells/cm^2^ for leukemia). Tested compounds (0.1–25 μg/mL) dissolved in 1% DMSO (100 µL) were added to each well and incubated for 72 h. Control groups received the same amount of DMSO. After 69 h of treatment, 25 μL of MTT (5 mg/mL) were added. Three hours later, the MTT formazan product was dissolved in 100 μL of DMSO, and absorbance was measured at 595 nm using a multiplate reader (Thermo Plate^®^, TP Reader, São Paulo, SP, Brasil) [28].

### 3.5. Cytotoxicity Assay on RAW 264.7

Murine peritoneal macrophages of the cell line RAW 264.7 were obtained from the American Type Culture Collection (ATCC, Rockville, MD, USA), and cultured at 37 °C with CO_2_ at 5% in DMEM-F12 supplemented with 10% fetal bovine serum (FBS; Hyclone^TM^, Logan, UT, USA) and gentamicin (50 μg/mL). RAW 264.7 cells (5 × 10^5^ cells/mL) were cultured and incubated for 24 h with the riparins A, B, C, D, E and F at the final concentration of 25 µg/mL for 48 h [29]. The cytotoxicity assay was performed by LDH assay. The release of LDH (cytoplasmic lactate dehydrogenase enzyme) was determined using 50 μL of culture supernatant with 100 μL of LDH substrate plus and 5 μL ferric alum (5 mg/mL), at 37 °C for 3 min. Then, 100 μL of the NAD solution and phenazine methosulphate were added to the mixture, maintaining the temperature at 37 °C for 5 min (Labrax Commercial Kit, Clontech, Palo Alto, CA, USA). LDH is an oxidoreductase which catalyzes the interconversion of lactate and pyruvate and, for being a stable cytosolic enzyme, after damage to cell membrane it is released in the cellular environment and because of this LDH is an indirect indicator of cytotoxicity. LDH concentration was determined by colorimetry at 492 nm. The specific release of LDH was calculated as percentage of the controls (not treated cells as negative control and cells treated with 1%Triton X-100 as positive control) [30].

### 3.6. Statistical Analysis

The obtained data were evaluated by one-way analysis of variance (ANOVA) followed by a Student Newman Keuls *post hoc* test. The data concerning release percentage of cytoplasmatic enzyme lactate dehydrogenase (LDH) were reported by mean ± standard deviation and evaluated by one-way analysis of variance (ANOVA) followed by a Tukey *post hoc* test. In all cases differences were considered significant if *p <* 0.05, the means and respective standard errors were analyzed in the Graph Pad Prism version 5.0 for Windows software (GraphPad Software Incorporated, San Diego, CA, USA). The IC_50_ values and their 95% confidence intervals for two different experiments were obtained by nonlinear regression using GraphPad Prism version 5.0.

## 4. Conclusions

The results of *in vitro* tests suggest that because of the removal capacity of hydroxyl free radical and nitric oxide, as well as by avoiding TBARS formation, the tested substances are promising biologically active compounds with antioxidant properties. Additional chemical, pharmacological and biotechnological studies must be performed in order to elucidate the viability of using these substances in the production of possible medicines, destined to combat free radicals, particularly in cancer. In cytotoxicity tests the samples of riparins C, D, E and F were active, which suggests a need for further investigation, as a significant cell growth inhibition potential for these substances is indicated. The mechanism of that process still needs to be elucidated in future tests.
